# Collaborative peer feedback in L2 writing: Affective, behavioral, cognitive, and social engagement

**DOI:** 10.3389/fpsyg.2023.1078141

**Published:** 2023-01-26

**Authors:** Wenxue Chen, Donghong Liu, Cancan Lin

**Affiliations:** School of Foreign Languages, Southeast University, Nanjing, China

**Keywords:** collaborative peer feedback, task engagement, peer interaction, L2 writing, collaborative learning

## Abstract

The recent two decades have witnessed a greater interest in L2 writing research to explore how individual learners engage with and participate in peer feedback. However, not much attention has been directed to peer feedback in the collaborative format, despite the fact that peer collaboration can enable learners to draw on their respective strengths and pool their knowledge. In this qualitative study, we adopted an educational psychological perspective to discover the intricate nature of learners working together to give anonymous feedback to their peers. In addition to learners’ cognitive engagement with the correction and revision process, we also investigated learners’ affective, behavioral, and social engagement in collaborative peer feedback. The findings show that, although learners can cognitively engage with the task by identifying a number of language-related problems and providing feedback, their affective, behavioral, and social engagement differed considerably. While some participants’ engagement was relatively extensive, especially in the affective and social aspect, others’ engagement was at a relatively limited level, characterized by negative emotions and low mutuality in peer interaction. The unpleasant task experience affected their attitudes toward collaborative peer feedback activities and their willingness to participate in subsequent tasks.

## Introduction

In the field of educational psychology, learner engagement is often seen as a crucial prerequisite for success in learning. It is defined as learners’ physical and psychological energies devoted to accomplishing a learning task (Fredricks et al., 2004; Schunk and Mullen, 2012). While physical energies refer to the behavioral efforts learners put forth to successfully complete the task, psychological energies embody the cognitive readiness and emotional state of the learner during task completion. Education researchers posit that learner engagement is a key determinant of learning outcomes and compare it to “*the holy grail of learning*” (Sinatra et al., 2015, p. 1), as it reflects not only students’ level of mental activation but also their endeavor to seek for and participate in different learning activities.

SLA researchers have adapted the concept of learner engagement to the L2 context by focusing on students’ engagement with the target language (Svalberg, 2009) or engagement in task-based interaction (Philp and Duchesne, 2016). In general, the available research has mostly followed Svalberg’s model of learner engagement, since it establishes a direct link between engagement and language learning. This is especially true among studies into peer feedback because this type of activity amplifies language use and attention to form (e.g., Han and Hyland, 2015; Zheng and Yu, 2018; Yu et al., 2019). However, while focusing on engagement with language, these studies have overlooked the communicative nature of the peer feedback task—a two-way information exchange task between the writer and the reviewer or among the reviewers. Students’ learning experience in tasks is shaped by their use of language as well as the task itself. Therefore, learners’ physical and psychological engagement with the task and engagement in interacting with their peers are of equal importance to their engagement with the target language. Drawing on Philp and Duchesne’s (2016) framework of task engagement, the present study investigated learners’ affective, cognitive, behavioral, and social engagement in an L2 writing task where learners work jointly to provide feedback. By contextualizing the different domains of engagement in collaborative tasks, the research aimed to clarify the mechanism shaping learner engagement and make a fresh attempt to address the peer collaboration process in feedback activities.

## Engagement as a multidimensional construct

Contemporary approaches to learner engagement have conceptualized it as a multidimensional construct, which has been mainly inspired by the model of Svalberg’s (2009) of engagement with language (EWL) and Philp and Duchesne’s (2016) model of task engagement (Engagement with the task, EWT). EWL is defined as “*a cognitive, and/or affective, and/or social state and a process in which the learner is the agent and the language is the object and may be the vehicle (means of communication)*” (Svalberg, 2009, p.244). The cognitive-affective-social trio focuses on learners’ attention to form which facilitates the construction of language awareness. It uncovers the multidimensional nature of engagement in the context of language learning and use. More recently, Philp and Duchesne (2016), also acknowledging the multifacetedness of engagement, proposed the model of EWT from an educational psychological perspective. EWT, situated in the context of task-based interaction, is defined as “*a state of heightened attention and involvement*” (Philp and Duchesne, 2016, p.3). It consists of four dimensions: cognitive, behavioral, social, and emotional. EWL and EWT are not mutually exclusive. While EWL focuses particularly on how cognitive, affective, and social factors influence learners’ attention to the target language, EWT considers learners’ experience in carrying out an L2 activity, including their attention to both language form and task content. In a sense, EWT is a more complex and dynamic model than EWL to explain the process and outcome of L2 learning. Another similarity between the two models is their acknowledgment of the overlapping and interdependent nature of these dimensions (Lambert et al., 2017; Nakamura et al., 2021). For example, a learner who is disinterested in a task (i.e., emotionally disengaged) is likely to be behaviorally off task (i.e., behaviorally disengaged). Philp and Duchesne also contend that emotional engagement is central to EWT because it links other facets and decides whether learner engagement can be activated.

A substantial number of empirical studies have been conducted to explore the interaction between different dimensions of engagement. When drawing on the model of EWL to explore how task complexity and task mode mediated learners’ attention to language form, Baralt et al. (2016) found that learners’ social relationships with partners and their affective responses toward the task and their partners can facilitate or impede cognitive engagement. That is, when learners were supportive and interactive and perceived the task as useful and fun, they demonstrated greater cognitive engagement with the target language. Zhang (2021), also adopting the model of EWL, focused on how three dyads’ cognitive engagement was influenced by social and affective dimensions of engagement in a collaborative writing task. It was found that dyads which formed a more collaborative relationship and perceived the task as useful identified more language-related problems and elaborated on them. By contrast, a non-collaborative dyad that had negative perceptions of the task missed many opportunities to expand on their linguistic items. Although Philp and Duchesne (2016) also claim that the four dimensions in their EWT model are interdependent, there is a lack of empirical research attempting to solve the mystery. The present study aims to bridge the gap by examining how cognitive, behavioral, social, and affective engagement interacts with one another to influence learners’ task experience.

## Conceptual framework of learner engagement in peer interaction

The conceptualization of learner engagement as a multifaceted and interdependent construct underpins the current study. In line with previous studies, we adapted the more complex engagement model by Philp and Duchesne (2016) which includes cognitive, behavioral, social, and emotional dimensions. The ways we operationalized the four dimensions are as follows.

Cognitive engagement refers to sustained attention, mental effort, and self-regulation strategies (Helme and Clarke, 2001). In SLA, the idea of language-related episode (LRE) has been proposed to capture learners’ attention to the target language in collaborative tasks (Swain and Lapkin, 1998). According to Swain and Lapkin, LREs are incidences where learners talk about the language they produce, question the usage of the target language, and correct themselves or others.

Behavioral engagement is described as being “on-task” (Philp and Duchesne, 2016). Commonly used measures involve the number of words and turns and the amount of time on task (Lambert et al., 2017; Phung, 2017; Qiu and Lo, 2017). Some scholars argue that this dimension overlaps with the other three dimensions. That is, learners demonstrate their cognitive, social, and emotional engagement through behavioral indicators. For example, when learners are cognitively engaged, they deliberate over language features and task content. Their cognitive engagement is manifested in language output which overlaps with the measures of behavioral engagement such as turns and words (Baralt et al., 2016; Zhang, 2021).

Social engagement is associated with social relationships between learners, manifested by the level of mutuality and reciprocity between interlocutors. Informed by Storch’s seminal work on patterns of interaction, Philp and Duchesne (2016) argue that when learners are socially engaged, “*they listen to one another, draw from one another’s expertise and ideas, and provide feedback to one another*” (p.10). Storch (2002) measured learners’ equality and mutuality in peer interaction to indicate their engagement with the task as well as with each other’s contribution. This approach has been employed by a large volume of research which looks into learners’ role relationship in collaborative dialog (e.g., Watanabe and Swain, 2007; Watanabe, 2008; Storch and Aldosari, 2013).

In Philp and Duchesne’s research, emotional engagement refers to learners’ enthusiasm, interest, enjoyment, and boredom during task completion. In the present study, we used the term “affective engagement” rather than “emotional engagement,” because we believe that emotional engagement provides only a partial picture of its dimension. It is affective engagement that reflects not only learners’ emotions displayed during interaction, but their attitudes and evaluations after task completion (Phung, 2017). These attitudes and evaluations will influence their subsequent performance in tasks of a similar kind, which eventually affect the L2 learning outcome. Following previous studies (Fan and Xu, 2020; Zhang, 2021), we subdivide affective engagement into two components: affect and value. Affect refers to learners’ feelings and emotions displayed during the collaborative feedback activity, whereas value refers to learners’ attitudes toward and evaluation of the value of the collaborative feedback activity.

## Empirical research on collaborative peer feedback in L2 writing

The effectiveness of collaborative peer feedback has been explained in studies which investigate collaborative dialog and L2 writing through reference to sociocultural theory and process writing theory. Sociocultural SLA researchers claim that the assistance learners provide with one another in peer interaction allows them to jointly achieve what they cannot achieve individually. When learners collaborate with each other, they acquire the target language and writing skills by deliberating about language choices, articulating uncertainties, and providing suggestions (Swain and Lapkin, 1998; Storch, 2002). Process writing theory views writing as a process of meaning-making and knowledge-building rather than a single written product. Peer revision is of paramount importance in this process because it creates “*opportunities for them (students) to discover and negotiate meaning, to explore effective ways of expressing meaning, to practice a wide range of language and writing skills, and to assume a more active role in the learning process*” (Hu, 2005, p. 322).

Numerous studies have detailed the benefits of collaborative peer feedback in L2 writing, including opportunities for noticing and uptake (De Guerrero and Villamil, 2000; Hyland and Hyland, 2006; Diab, 2010), awareness of text and audience (Tsui and Ng, 2000; Berggren, 2015), and improvement in writing strategies and skills (Liu and Hansen, 2002; Hu, 2005; Min, 2005). They tend to agree that peer revision activities enable learners to focus on the use of the target language and text quality, so that they can make an interpsychological effort to achieve intersubjectivity. Researchers have also identified some features of collaborative peer feedback. When learners read their peer’s writing, they are more likely to attend to the global aspects of language use, such as organization, content information, and cohesion, than local aspects such as vocabulary and grammar (Lundstrom and Baker, 2009; Berggren, 2015). The common strategies they use to provide feedback include the use of L1 and advising and requesting clarification (Villamil and De Guerrero, 1996). To date, L2 writing scholars have pointed out two key factors that may influence students’ performance in feedback activities. First, the social relationship learners form has a direct relationship with their linguistic gains. Researchers have found that a collaborative relationship facilitates language-related discussions among peers while an authoritative stance inhibits productive corrective feedback (Zhu and Mitchell, 2012; Fan and Xu, 2020). Second, student motives play a significant role in guiding their participation in peer feedback. Yu and Lee (2015) found that students’ motives for participating in peer revisions, especially their perceptions about this type of task and previous experience in carrying out the activity, influence and interact with the stance and role relationship they adopt in the task which further impact on their revisions. When they perceive peer feedback as a valuable source of improving L2 writing, they tend to collaborate with fellow students and deliberate over linguistic issues in the text.

In spite of the influx in peer feedback studies, an overwhelming majority looked into the collaborative work between the writer and the feedback giver. Although the dialog between the author and the reviewer allows for direct negotiation, such communication discourages critical comments. A few studies have reported learners’ reluctance to disagree with their peers and criticize their work as a result of face saving in an author-reviewer interaction mode (Nelson and Carson, 1998; Kamimura, 2006). For Asian students, the interpersonal harmony may be more important than offering their peers constructive criticism (Wang, 2014; Chang, 2016). Alshuraidah and Storch (2019) believe that having learners provide feedback in pairs without the presence of the text author would encourage free share of thoughts and comments. More importantly, with both participants endeavoring to give feedback, there would be more active elicitation of reviewer comments. The researchers compared the nature and quality of collaborative peer feedback with that of individual feedback. They found more feedback points in pairs than among individuals. The pairs also provided their peers with more negative comments. In terms of quality, the negative comments contained more constructive suggestions than the positive comments. Alshuraidah and Storch thus argue that collaborative peer feedback creates opportunities for learners to draw on their respective strengths and collectively resolve their uncertainties. It involves questioning, discussing, and explaining to their peers rather than simply making individual decisions. In the interview, the research participants also reported positive attitudes toward collaborative peer feedback, as it guaranteed the appropriateness of their correction. This study offers another approach to operationalize collaborative peer revision.

In the present study, we have the reviewers work collaboratively to give feedback to an anonymous written text by their peer. When comparing the effects of anonymous and identifiable peer feedback on L2 learners’ writing, Zaccaron and Xhafaj (2020) have shown that learners feel more comfortable when giving anonymous peer feedback, because it diminishes their fear of giving too many corrections which may also contain erroneous feedback. On the other hand, although identifiable peer feedback help clarify outstanding feedback issues, it arouses some hostility between authors and reviewers and also among reviewers. The current research aimed to see whether learners can engage in anonymous peer feedback task and its potential in encouraging L2 learning. In addition to learners’ cognitive engagement with the correction and revision process, we also investigated learners’ affective, behavioral, and social engagement in collaborative peer feedback. This multifaceted model allows us to include an emphasis on both attention (the cognitive dimension) and the affective, behavioral, and social dimensions that support effective learning. Specifically, the following research questions guided the current study:

1. How did the students engage with collaborative peer feedback affectively, behaviorally, cognitively, and socially?
2. How did the four dimensions of engagement interact with one another to influence the learners’ task experience?

## Methods

### Research context and participants

The qualitative study was carried out at a comprehensive university in Eastern China. The 16 volunteering participants came from the same English class and were all majored in Japanese language. There were 7 males and 9 female students. They were between 18 and 19 years old and were in their second semester of the second year when the data were collected. They had two sessions of 90-min class each week for 16 weeks. While the first-year English class focused on listening and speaking skills, the second-year study aimed to improve the students’ reading and writing proficiency. In the second semester, the learners were taught how to compose argumentative essays, including text structure, grammar, and useful expressions. During the class, they had sufficient experience of peer collaboration but did not have any chance to do collaborative peer revision. However, the class teacher tended to guide them to discuss their peers’ writings and identify the strengths and weaknesses. According to the teacher’s record, all participants had passed the college English Test Band 6 (CET 6)—a nationwide English proficiency test. The exam results placed them at the intermediate level, ranging from lower intermediate to upper intermediate.

### Data collection

The data collected include the recordings of students’ interaction in giving feedback, their corrections and comments, and the retrospective interview with 8 of the 16 participants. In week 1, the participants were asked to self-select their working partners and were told that their task performance would be recorded. All participants signed the consent form and agreed to be video-recorded. In week 2, they wrote an essay to explain the reason for young people spending less time on weekends to do outdoors activities and to provide suggestions to encourage them to go out. The participants were asked to write about 250 words and were given 45 min to complete the task. In week 3, they worked in pairs to give feedback to an anonymous essay on the same topic written by students from another class taught by the same teacher. They were given 30 min to carry out the feedback task. The students were not forced to use English to interact with each other, because the feedback task aimed to encourage complex metalanguage which can lead to more and richer feedback (Zaccaron and Xhafaj, 2020). The peer interaction data were transcribed and coded in the following weeks. In week 6, 8 out of the 16 participants volunteered to attend the retrospective interview. Other participants declined to be interviewed because of personal reasons or time issues. Table 1 displays the data collection procedure.

**Table 1:** Data collection procedure.

Time	Procedures
Week 1	Self-select a working partner and Sign the consent form
Week 2	Write an essay in 45 min individually
Week 3	Offer feedback to an anonymous essay collaboratively
Week 4 and 5	Data transcription and coding
Week 6	Retrospective interview

The individual retrospective interviews were conducted to provide evidence for affective engagement and to analyze the interaction between cognitive, behavioral, social, and affective dimensions. In a retrospective interview, the interviewees are questioned regarding what they have experienced in the past. This method has been used to address a wide range of issues in L2 research, including L2 writing and collaborative dialogs (e.g., Watanabe and Swain, 2007; Watanabe, 2008; Chen and Yu, 2019; Chen and Lee, 2022).

The interview fell into two parts. It started with a stimulated recall by playing some episodes of the video recording and asking pertinent questions to the participant, in order to understand the student’s thoughts and feelings and check whether there was a mismatch between the researchers’ interpretation and the student’s actions. The purpose of adopting stimulated recalls in this study is to seek explanations for particular behaviors so as to better understand the nature of learners’ interaction and their engagement with the task. In the second part, the interviewers raised some general questions about the learner’s task experience and his/her perceptions of the task and the partner, which would generate evidence of his/her engagement.

### Data analysis

The data analysis followed the multidimensional model of task engagement and consisted of four parts—cognitive engagement, behavioral engagement, social engagement, and affective engagement.

#### Cognitive engagement

**Figure 1:**
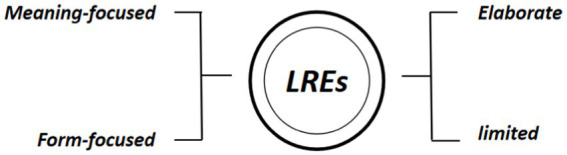
Categorization of LREs.

Cognitive engagement refers to learners’ heightened attention to the target language in task. Recent research has recognized the need to measure cognitive engagement from both learners’ discussion of language features and their attention to meaning to depict learner engagement in collaborative tasks (Dao and McDonough, 2018; Dao, 2019; Dao et al., 2021). In this study, we followed previous approach by identifying the number of LREs and further categorized them into meaning-based LREs School of Foreign Languages, and form-based LREs. While meaning-based LREs center on global aspects of the essay such as text organization and cohesion, form-based LREs are more concerned with local aspects such as word choice and grammar. The LREs were further categorized as either elaborate or limited. An elaborate LRE involves the participants’ joint efforts while limited LREs lack contribution by one of the interlocutors. Figure 1 summarizes the different categories of LREs classified. Appendix 1 provides two coding examples to illustrate different categories of LRE.

#### Behavioral engagement

Behavioral engagement is usually regarded as the amount of time learners actively involve in the task. Although the participants were given 30 min to complete the task, most of them spent more time on it. In addition to time on task, we further operationalized behavioral engagement as turns and words produced to represent the behavioral efforts learners made in interacting with partners. In the interview, we also asked the participants about their perceptions of the time for task completion, which was used as supplementary evidence to indicate learners’ behavioral engagement.

#### Social engagement

*S*ocial engagement emphasizes the reciprocal nature of peer interaction. In the present study, it was measured by the patterns of interaction learners demonstrated in dialog. Storch (2002) pointed out four patterns: collaborative, expert/novice, dominant/dominant, and dominant/passive. While learners in collaborative and expert/novice patterns of interaction are willing to share ideas and negotiate meaning with each other, learners forming a dominant/dominant relationship and the dominant learner in the dominant/passive relationship tend to ignore their partners’ utterances. Features that helped distinguish among these patterns include the amount of individual contribution to task, the number of requests and questions, explanations, and suggestions, as well as the frequency of individualized decision-making and disregarding of partners’ utterances. In our data, one dyad did not fit into the four patterns proposed by Storch. In this dyad, one participant was devoted to the task but did not show any willingness to interact with his partner, despite the other interlocutor’s constant invitations for communication. As a result, they agreed to divide the text and each took responsibility for their own part of the text. This pattern of dialog resembles what Tan et al. (2010) called the cooperative pattern in which learners contribute different elements to achieve the task goal and fail to engage with each other’s contribution. Following Tan et al. (2010), we labeled this dyad as “cooperative” which showed low social engagement in the task.

#### Affective engagement

As mentioned previously, measurement of affective engagement consists of learners’ feelings and emotions during the task completion process (affect), and their perceptions and evaluations of the task (value). To gauge learners’ affective engagement, we looked for evidence from both the interaction data and the retrospective interview. During the task, learners’ expressions of their feelings and paralinguistic features such as laughter were coded as evidence. To ensure validity, these codings were checked with the participants in the stimulated recall interview. We had three deductive codes for affective engagement: (1) feelings and emotions when giving feedback; (2) interest and willingness in participating in the task; and (3) perceived value of the task. Learners’ expressions containing these codes were categorized and calculated in terms of frequency. For example, an interviewee regarded the feedback activity as a pleasant experience. The word *pleasant* was coded as an instance of positive evidence for feelings and emotions. In comparison, one participant felt embarrassed when working with his partner. In this case, *embarrassed* was coded as an instance of negative evidence for feelings and emotions. Table 2 summarizes the criteria and indicators for the analysis of the different dimensions of engagement.

**Table 2:** A summary of the analysis of EWT.

Dimension	Data source	Operationalization
Cognitive engagement	Peer interaction data^*^	Form-based LREs or meaning-based LREs Elaborate LREs or limited LREs
Behavioral engagement	Peer interaction data^*^ and the retrospective interview	Time on task Turns Words
Social engagement	Peer interaction data^*^ and the retrospective interview	Patterns of interaction: individual utterances requests and questions disregarding partner’s requests questions explanations suggestions individualized decision-making
Affective engagement	The retrospective interview^*^ and peer interaction data	Affect: Feelings and emotions when giving feedback; interest and willingness in participating in the task Value: Perceived value of the task

Data sources with ^*^ were the main sources for analysis of the corresponding dimension while others were used as supplementary materials.

To ensure inter-coder reliability, the peer interaction data including analysis of the LREs, patterns of interaction, conversation turns, word count, and time spent on task completion were coded by at least two researchers in this project. The three researchers did the coding in the first 10-min episode of a dyad, and then had a discussion and adjusted understanding of each criterion. After that, they did coding, respectively, to finish all the work. The initial inter-coder reliability for LREs and patterns of interaction were 89% and 87%, respectively. Discrepancies were then discussed until total agreement was reached. The inter-rater reliability for conversation turns, word count, and time spent on task completion ranged from 97% to 99%. For the interview, 20% of the data were jointly coded by the first author and the third author while the remaining were coded individually. Inter-coder agreement was 92%.

## Results

### Cognitive engagement

Learners’ cognitive engagement with the collaborative feedback activity was examined in terms of meaning-based and form-based LREs generated by peer dialog. A total number of 209 instances of language-related discussions were identified from learners’ dialogs. Table 3 shows the results concerning the number of LREs produced by each dyad. Of the 8 dyads, the first four pairs in Table 3 produced an overwhelming number of meaning-based LREs as opposed to the form-based, two directed a greater deal of attention to language form, and the last two dyads paid roughly equal attention to form and meaning. The results failed to illustrate any clear tendency for learners’ choice of language focus, but it did suggest a great difference in the number of LREs across dyads.

**Table 3:** Results for form-based and meaning-based LREs.

Dyad	Form-based LREs		Meaning-based LREs		Total
	*N*	%	*N*	%	*N*
Chen and Yue	1	7.14%	13	92.86%	14
Han and Wu	6	33.33%	12	66.67%	18
Zhang and Fu	8	34.78%	15	65.22%	23
Ru and Jun	6	40.00%	9	60.00%	15
Jin and Su	10	66.67%	5	33.33%	15
Yin and Yang	41	75.93%	13	24.07%	54
Li and Ze	16	43.24%	21	56.76%	37
Yan and Zhi	18	54.55%	15	45.45%	33

**Figure 2:**
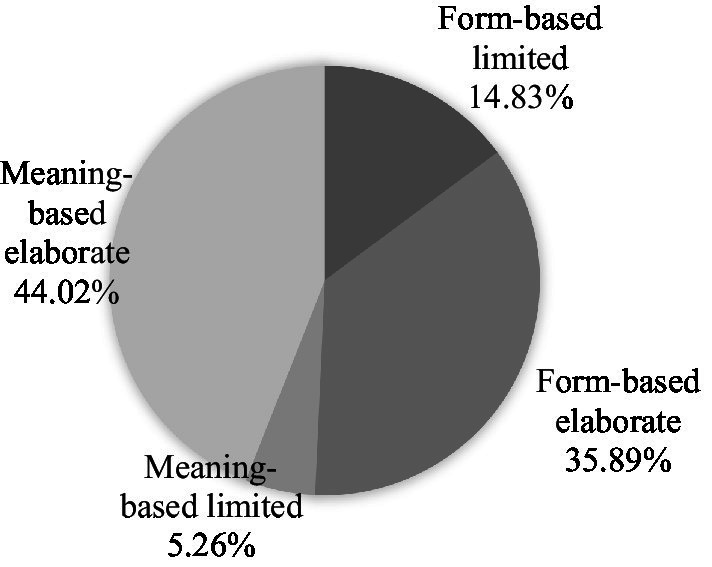
Distribution of LREs.

To determine the level of cognitive engagement, we further categorized the LREs as limited and elaborate. Figure 2 and Table 4 summarize the results for this analysis. In terms of form-focused LREs, the majority of the instances were elaborate, accounting for 35.89% of the total. However, four dyads tended to make joint efforts to expand their discussion over local aspects of the target language, while the other four were more likely to solve linguistic problems individually.

**Table 4:** Results for elaborate and limited LREs.

Dyad	Form-based LREs		Meaning-based LREs		Total
	Limited	Elaborate	Limited	Elaborate	
Chen and Yue	1	0	3	10	14
Han and Wu	4	2	3	9	18
Zhang and Fu	2	6	0	15	23
Ru and Jun	3	3	0	9	15
Jin and Su	9	1	5	0	15
Yin and Yang	5	36	0	13	54
Li and Ze	2	14	0	21	37
Yan and Zhi	5	13	0	15	33

Similarly, most of the instances of meaning-focused LREs were elaborate, accounting for 44.02% of the total. In 7 pairs, the number of elaborate discussions was much greater than that of limited LREs, which indicates that these participants could elaborate on the meaning-oriented issues they noticed during the task. Specifically, of the seven pairs, five always scrutinized the language issues they discovered. However, in Jin and Su’s dialog, not only was the number of LREs quite limited, but these instances were limited in terms of the level of scrutiny.

Overall, these results show that when completing the collaborative feedback task, learners were able to cognitively engage with both language meaning and form and most of the dyads could make joint efforts to deliberate over LREs.

### Behavioral engagement

We examined learners’ behavioral engagement with the collaborative feedback activity through three indicators: the amount of time on task, the number of turns, and word count. These indicators represent learners’ behavioral engagement in both reviewing the composition and interacting with partners.

Table 5 presents the results for these indicators. In terms of time on task, 7 out of 8 pairs spent more than the allocated time on this task. Although they were given 30 min to carry out the task, the average time for task completion reached 43 min. In the interview, all the eight interviewees stated that 30 min was not enough. As Yan said, “*We ran out of time, but we still wanted to ensure that we could find as many problems as possible to make the essay a better one*.” Han also admitted that he needed more time to focus on local aspects of the composition, although he completed the task with his partner within 30 min.

**Table 5:** Results for behavioral engagement.

Dyad	Time (min)	Turns	Words	Mean turn/min	Mean word/turn
Chen and Yue	35	127	3,222	3.63	25.37
Han and Wu	27	89	4,338	3.30	48.74
Zhang and Fu	51	300	4,763	5.88	15.88
Ru and Jun	38	101	1,947	2.66	19.28
Jin and Su	47	124	1,989	2.64	16.04
Yin and Yang	42	412	4,515	9.81	10.96
Li and Ze	52	386	4,321	7.42	11.19
Yan and Zhi	52	313	6,240	6.02	19.94

Regarding turns and word count, four dyads produced more than or equal to 300 turns which in turn generated a high word count. Clearer results can be found when we compare the means. Although the pairs of Han and Wu and Chen and Yue had less exchange of turns (3.30 and 3.63), they produced a considerable number of words during the task, which means each turn was at a greater length (48.74 and 25.37). By contrast, the other two pairs (Jin and Su and Ru and Jun) generated the lowest mean number of turns (2.64 and 2.66), although the time they spent was not the shortest. This indicates their low productivity and lack of communication with each other. Another pair worth noting is Yin and Yang who produced the most turns per minute (9.81) but shortest as well (10.96).

To conclude, according to the results of the three indicators, six dyads were behaviorally engaged, whereas the other two demonstrated greater behavioral engagement in reviewing the composition than in communicating with partners.

### Social engagement

Learners’ social engagement with the collaborative feedback activity can be manifested in the patterns of interaction they displayed. Table 6 presents the patterns of interaction across the eight dyads. Six dyads interacted in a collaborative pattern in which learners contributed jointly to reviewing the essay and engaged with each other’s contribution. Five learners in the six dyads were interviewed. They all agreed that they formed a collaborative relationship with their partners during the task. In the retrospective interview, Yin said that neither of them dominated the task and they supported each other. One dyad, Ru and Jun, displayed an expert/novice pattern, as Jun actively encouraged Ru to express her ideas and offered assistance to Ru throughout the task. In the interview, Ru mentioned a trait that Jun exhibited,

**Table 6:** Results for social engagement.

Dyad	Patterns of interaction
Chen and Yue	Collaborative
Han and Wu	Collaborative
Zhang and Fu	Collaborative
Ru and Jun	Expert/novice
Jin and Su	Cooperative
Yin and Yang	Collaborative
Li and Ze	Collaborative
Yan and Zhi	Collaborative

“Even though he was more competent than me, he never directly objected to my opinions. Initially, I was too discouraged and I even didn’t know what I should do. However, he encouraged me constantly and helped me when I had difficulty in expressing my opinions. Thus, I became gradually confident, and I could say more and contribute more to the revision.”

By contrast, Jin and Su worked in a cooperative manner. There was little negotiation in which both of them were actively involved. In the interview, both of them admitted that they failed to collaborate with each other. Overall, the results indicate that one dyad displayed low social engagement in this task, while others were socially engaged.

### Affective engagement

Learners’ affective engagement with the collaborative feedback activity was reflected in their feelings and emotions displayed during the activity as well as their attitudes toward and evaluation of the activity. Table 7 demonstrates the frequency of the interviewees expressing their positive and negative affects.

**Table 7:** Results for affective engagement.

	Feelings and emotions	Interest and willingness	Perceived value
Positive instances	20	26	35
Negative instances	6	5	10

Learners generally displayed positive feelings and emotions during the activity. This can also be exemplified by the frequent occurrence of laughter in some pairs’ interaction. A noteworthy case is that Jun teased about Ru in an unfriendly way during the activity by saying “*you are not useless at all*.” In the interview, both of them agreed that the interaction was enjoyable. Ru stated that “*I do not mind the jokes he made at all*” and “*I felt happy when I worked with my partner to revise the essay*.” Additionally, some other students also expressed their happiness and enjoyment. Han commented in the interview, “*It’s more fun when you work with a partner. You can exchange ideas with him. I prefer this kind of atmosphere*.”

However, two learners, Jin and Su, explicitly expressed disappointment and dissatisfaction during the activity. Jin complained about the composition and the task from time to time, such as “*I’m fed up with this essay*” and “*I’ve never heard that one can write an essay with others in an exam*.” Likewise, Su assessed the essay as one written by a junior high school student. In the interview, Jin emphasized that he felt unhappy and restless when completing the task. Interestingly, Su mentioned that his negative emotions were triggered by Jin’s reluctance to engage in collaboration.

With regard to learners’ attitudes and evaluation, 6 out of 8 learners interviewed had positive attitudes toward the activity and perceived it as a useful and valuable activity. They regarded the activity as an opportunity to learn from the merits and weaknesses of other students’ essays and to reflect on their own shortcomings. More importantly, they appreciated the value of collaborating with peers. Yin’s comments illustrated,

“I hadn’t noticed that the essay had a poor logical flow until I discussed it with my partner. Only through exchanging ideas with my partner, did I have the chance to reconsider the essay or some ideas in it. When working together, we could pay more attention to details and revise the essay more thoroughly.”

In addition, three learners also mentioned the psychological support offered by their partners. As Jun said, “*the support she gave to me was not only associated with writing skills, but was a kind of trust that made me feel assured*.” His partner, Ru, said, “*I felt more confident when I worked with my partner, because I cannot trust my ability*.” Given the usefulness and value they perceived, 6 learners expressed their preference and willingness to perform a collaborative task in the near future.

In comparison to these positive comments, Su and Jin’s attitudes were opposite. In the interview, Su said that he preferred to complete the task individually, since he believed that individual work could guarantee the coherence of the essay. His partner, Jin, could not perceive any benefits of the activity. What’s more, he raised a concern about the efficiency of reviewing essays with peers. It should be noted that both of them expressed their unwillingness to participate in collaborative peer feedback in the future.

To sum up, most learners showed great affective engagement with the collaborative feedback activity, as they felt agreeable and relaxed during the activity and could perceive its value. One dyad, Jin and Su, was not as affectively engaged as other learners, given the negative emotions and perceptions they expressed.

### Interaction between cognitive, behavioral, social, and affective dimensions

**Figure 3:**
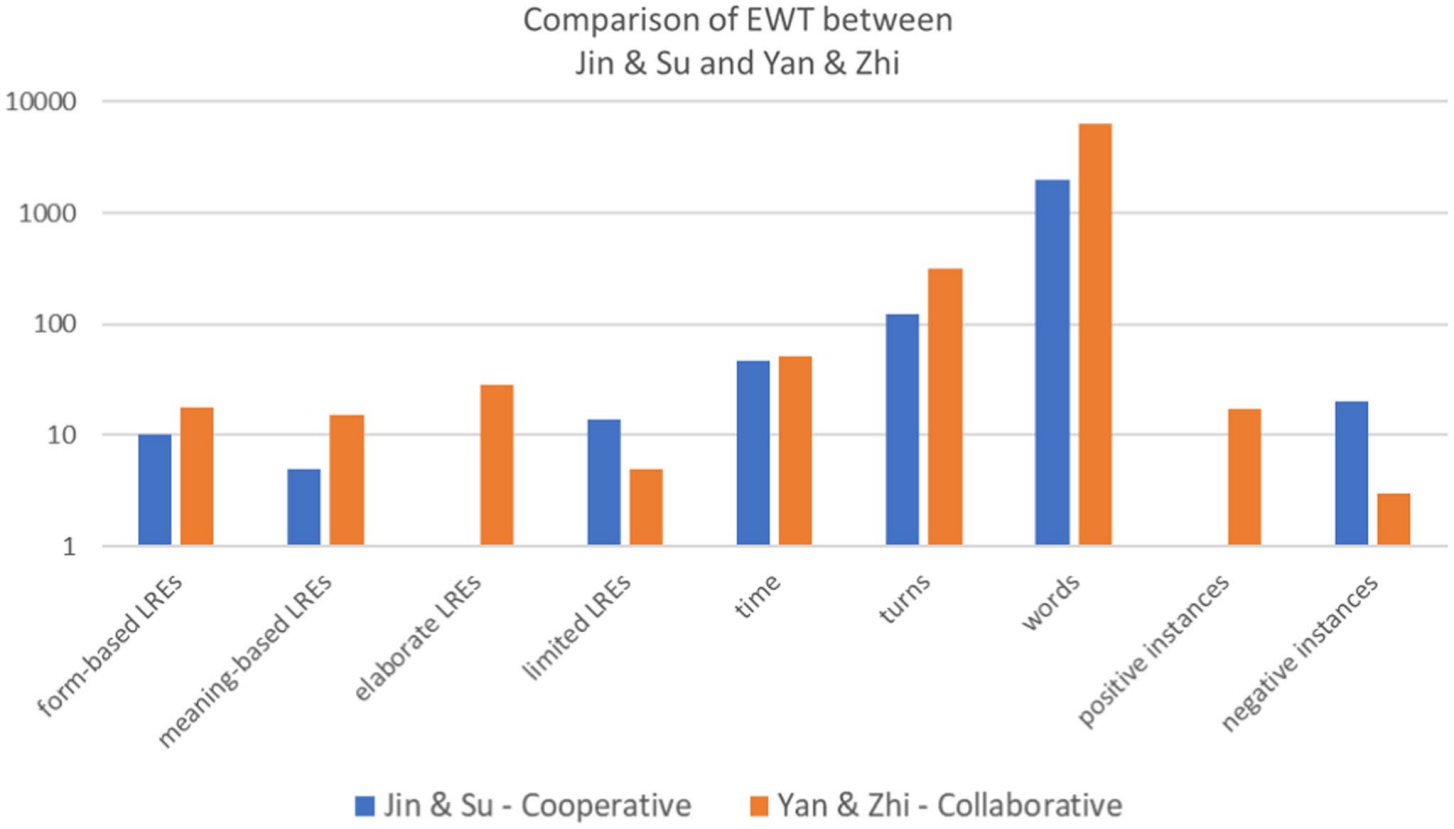
Comparison of EWT between two pairs.

To illustrate the interaction between the four dimensions, we selected two pairs, Jin and Su and Yan and Zhi, since they displayed strong contrasting pictures when completing the task, as shown in Table 8 and Figure 3.

**Table 8:** Comparison of EWT between Jin and Su and Yan and Zhi.

Dimension	Measure	Jin and Su	Yan and Zhi
Cognitive engagement	Form-based LREs	10	18
	Meaning-based LREs	5	15
	Elaborate LREs	1	28
	Limited LREs	14	5
Behavioral engagement	Time	47	52
	Turns	124	313
	Words	1989	6,240
Social engagement	Patterns of interaction	Cooperative	Collaborative
Affective engagement	Positive instances	1	17
	Negative instances	20	3

In Jin and Su’s case, negative attitudes toward this activity undermined their willingness to collaborate with each other. As the task proceeded, the low level of social engagement led to the participants’ destructive emotions and they became independent of each other without any collaboration. Finally, the pair did not generate sufficient turns and language-related negotiations.

Jin had negative attitudes toward collaborative peer feedback and was dissatisfied during the task, as he complained a lot. When Su made efforts to involve him in negotiation, he ignored the invitation and focused on independent thinking. According to Su’s self-report, as all these attempts failed, he turned a bit upset and angry. Consequently, in the middle of the task, he suggested dividing the essay into two parts and each of them revised their own part individually. The lack of social engagement further influenced their behavioral engagement with the dialog, which was reflected in a small number of turns and words. Additionally, the non-collaborative relationship also affected their cognitive engagement, as most of the LREs they produced were limited. That is, language-related issues were often initiated and solved by one of them without joint deliberation, as shown in Excerpt 1.

**Excerpt 1:**
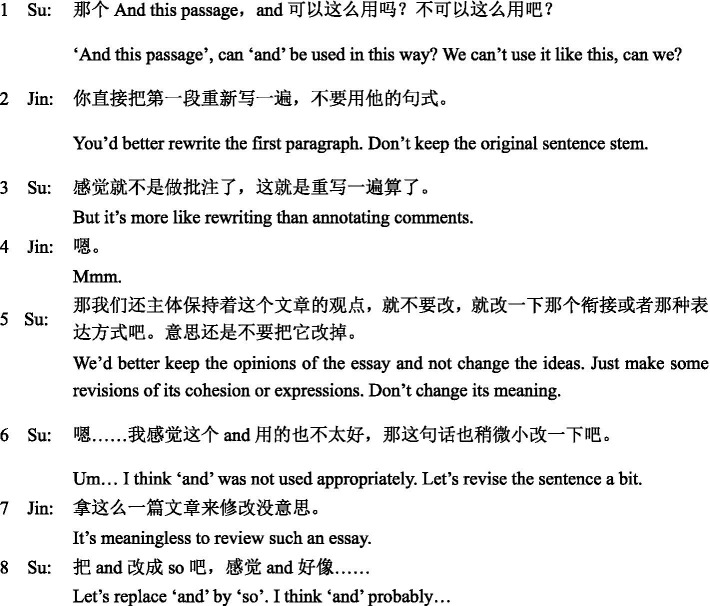


In this excerpt, Su initiated a question about the use of “and” at the beginning of a sentence. Instead of offering a direct answer, Jin suggested rewriting the whole paragraph. Su expressed his disagreement (turn 5), but he did not receive any response from Jin. In turn 6, he invited Jin to revise the sentence again. Jin complained about the essay (turn 7), rather than negotiating with Su to find an appropriate solution. The episode ended with Su’s monolog (turn 8). This excerpt illustrates that they were unable to expand their negotiation over language-related problems and make resolutions jointly.

A clear contrast was found in the other pair, Yan and Zhi. Affectively, they had a positive disposition toward the task, which facilitated the formation of a collaborative relationship. When collaborating with each other, they affectively engaged more. Consequently, the high level of affective and social engagement contributed to greater behavioral and cognitive engagement.

Yan and Zhi were nervous at the beginning of the task, as Yan reported in the interview. Different from Jin, Yan held positive attitudes toward working with peers. She stated that she trusted more in joint efforts than individual work. The positive perception was reflected in her attempts to actively negotiate with Zhi. Influenced by their positive affective states, a collaborative relationship was gradually built between them. In the interview, Yan said that the collaborative relationship made them less stressed and more relaxed. This can be triangulated by a high frequency of laughter in their interaction. It suggests that a high level of social engagement could in turn positively contribute to learners’ affective engagement. The great affective and social engagement further facilitated their behavioral engagement. Not only did they spend additional time revising the essay, but they were highly engaged in interacting with each other, as they produced a sizeable number of turns and words. Similarly, the high level of affective and social engagement also led to greater instances of cognitive engagement. A majority of language-related issues were jointly deliberated, as illustrated in Excerpt 2.

**Excerpt 2:**
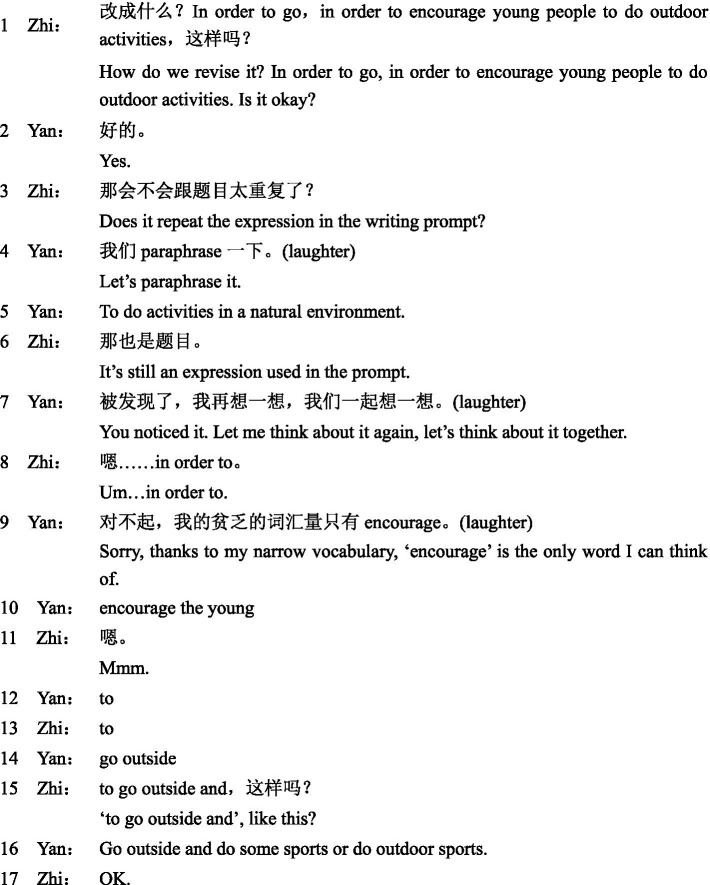


In this excerpt, Yan and Zhi were considering adding a clause to the third paragraph of the essay. Zhi initiated an option and found it not appropriate (turn 1 and turn 3). In the following turns, they mulled over this issue and pooled their resources to resolve it. Their laughter continued over several turns (turn 4 to turn 9). Interestingly, in turn 7, Yan rephrased her suggestion by changing “let me” to “let us.” The choice of first-person plurals reflects joint ownership of the task and shared responsibility for task completion (Donato, 1988; Villamil and De Guerrero, 1996; Storch, 2001). In contrast to Jin’s repeated ignoring of his partner’s request, Yan in this excerpt shows active participation, attentive listening, and conscious awareness of peer collaboration. Although both excerpts were coded as form-based LREs, one was limited without any peer support whereas the other was elaborate and resulted in a successful resolution.

## Discussion

By adapting Philp and Duchesne’s (2016) EWT framework, this study investigated how Chinese EFL learners engaged with a collaborative peer feedback task. It initiated two major research questions. One was the level of learner engagement in anonymous peer revision. The second research question explored the interaction among the different dimensions of learner engagement.

The results revealed that a majority of these intermediate adult learners were actively engaged in the task. Cognitively, the students were able to notice a range of problematic language-related items in their peers’ essays. In opposition to previous findings that intermediate learners usually direct more attention to meaning than form in peer feedback (Lundstrom and Baker, 2009; Berggren, 2015), the students in our study seemed to prioritize meaning or form at random. In the interview, some expressed great concern about the presentation and organization of ideas, whereas others considered vocabulary and grammar as the major weakness in their allocated essay.

Although the dyads differed from one another in terms of the foci of LREs (meaning vs. form), they tended to elaborate on most of the LREs in order to make joint decisions. The finding here lends more support to this format of peer feedback. In comparison to the author-reviewer collaboration in which the reviewer individually decides the language focus and points out the error, collaborative peer feedback allows learners to tentatively initiate their concerns and negotiate with each other to confirm the problem and provide correction, with both members invested in this endeavor. Alshuraidah and Storch (2019) also pointed out the potential weakness of lacking counter-suggestions in traditional peer feedback activities, and thus called for attention to collaborative feedback among reviewers. When we manipulate task design to encourage collaboration and interaction, the ultimate goal is to maximize L2 deliberations, as we tend to believe that two heads are better than one. In this regard, the interaction between/among reviewers has a comparative advantage over the author-reviewer mode of communication.

The results for behavioral engagement indicated that the participants were generally on task. Most of them spent more than 30 min on the task. However, their contribution to the collaborative dialog differed markedly. While some spent their task time sharing thoughts with partners, others preferred to use the time for independent thinking. This finding uncovered two facets underlying behavioral engagement in collaborative activities: engagement with the task and engagement with the dialog. Those who devoted themselves to the task and required additional time for completion showed a high level of task engagement, but they can be disengaged in the communication with their fellow partners. Such behaviors may undermine the benefits that peer collaboration can bring to task success and to individual students. In fact, the dimensions of behavior and social relationship are interwoven here, as learners are more likely to participate in peer interaction when they form a collaborative relationship.

When measuring learners’ social engagement, we counted not only their utterances but their amount of effort to engage with each other’s production. Seven out of 8 pairs were interactive and supportive. They co-constructed the dialog by frequent requests and questions and extension of each other’s talk. Previous studies into L2 writing have detailed how collaborative and expert/novice patterns facilitate the generation of LREs (e.g., Kim and McDonough, 2008; Storch, 2008; Chen and Yu, 2019). In the present study, learners forming a collaborative relationship produced LREs of higher quantity and quality than those employing expert/novice and cooperative patterns. When socially engaged, learners were more willing to unravel complex linguistic issues as they became empowered by their supportive peers. Conversely, learners in a cooperative interaction manner missed many opportunities to negotiate meaning and form with each other. As a result, they turned the collaborative task into an individual one with monologs and individualized decision-making.

In the pair of Jin and Su, Jin’s utter disregard of Su’s invitation for dialog led to Su’s disappointment over collaborative tasks. In the interview, he perceived the task as an inefficient way to revise text. Throughout the task completion process, these emotions and perceptions were intertwined with their role relationship in this dyad. The interaction between social engagement and affective engagement has been demonstrated by a few studies. Some found an impact of social relationships on affective engagement and claimed that low affective engagement undermined interpersonal relationships (Zhang, 2021), while others held that peer exclusion in collaborative dialog can result in negative emotions and deliberate disengagement (Baralt et al., 2016). Philp and Duchesne (2016) even claim that affective engagement is the key to determine the level of social, behavioral, and cognitive engagement. In the current research, we identified not only the interaction between the two dimensions and its effect on behavioral and cognitive engagement, but the joint effect of the two dimensions on learners’ willingness to participate in future tasks. The collaborative and expert/novice pairs all expressed enthusiasm for participating in similar tasks in the future, the cooperative dyad, however, preferred to do this type of task alone. The interviewers also asked members of the cooperative dyad whether they would give it another try by working with a different partner. Both declined. Since it was the first time these students carried out the collaborative peer feedback task, their social experience and attitudes toward this task type may have long-term influence on their L2 learning. From this perspective, the influence of social and affective engagement should not be limited only on the amount of LRE production in a one-off task. Rather, the influence is profound and has far-reaching consequences.

## Conclusion

The study, adopting a qualitative approach, advances previous research on peer feedback by drawing on the EWT model of Philp and Duchesne (2016) to examine the cognitive, behavioral, social, and affective dimensions of engagement in a collaborative task. We believe that the EWT framework is better suited to analyze learners’ participation in peer interaction because it recognizes the complexity of peer collaboration, which is not only a way to encourage attention to language but also a source of peer support. In line with previous studies on learner engagement (Baralt et al., 2016; Lambert et al., 2017; Nakamura et al., 2021; Zhang, 2021), the results revealed the interwoven and interdependent nature of the four dimensions. In particular, those who demonstrated high social engagement and perceived benefits of peer collaboration tended to be increasingly engaged with the task in the cognitive and behavioral aspects. By contrast, negative emotions and perceptions of the task and the non-collaborative interaction mode led to a withdrawal from interaction, which, in turn, impeded the identification and resolution of LREs. More importantly, these dimensions interact with each other to shape learners’ task experience which consequently affected their attitudes toward collaborative peer feedback activities and their willingness to participate in subsequent tasks.

These findings suggest that an anonymous collaborative peer feedback activity is able to reduce learner anxiety and encourage reciprocal feedback. Without the presence of the writer, the reviewers can speak their minds more openly and freely. In Alshuraidah and Storch’s (2019) study, the collaborative reviewers provided more negative comments than positive ones, although their personal information was not anonymous. In the present research, we did not identify any positive comments by the 8 pairs. According to previous findings, negative feedback can elicit more critical and constructive feedback and result in a higher uptake rate (van den Bos and Tan, 2019; Zaccaron and Xhafaj, 2020). Therefore, we call for more research into this relatively infant area and more attention to this type of peer feedback as a useful technique in the L2 writing classroom. Moreover, as a small-scale study contextualized in a writing class, the present research also welcomes future research to employ a quantitative approach with more L2 learners and in diverse L2 settings to understand this type of peer feedback for the better.

The results also inform both SLA researchers and education practitioners that learners’ affective and social engagement has long-term influence on L2 teaching and learning. For researchers, longitudinal studies can be conducted to explore whether learners could change their perceptions of the task and role relationship with their partner given more practice. For teachers, learners’ foretaste of a new task will have a cumulative impact on their attendance and engagement in similar tasks. The design and manipulation of the initial task should be carefully considered in order to increase the level of affective and social engagement.

## Data availability statement

The original contributions presented in the study are included in the article/supplementary material, further inquiries can be directed to the corresponding author.

## Ethics statement

The studies involving human participants were reviewed and approved by the Ethics Committee of Southeast University. The patients/participants provided their written informed consent to participate in this study.

## Author contributions

WX: data collection, data transcription and analysis, and paper writing. DL: data analysis and paper writing and revising. CL: data analysis and paper revising. All authors contributed to the article and approved the submitted version.

## Funding

This work was supported by the National Social Science Funds (Grant No. 21FYYB016), Fundamental Research Funds for the Central Universities (Grant No.2242021S20025), and Curriculum Reform Funds for the Central Universities (Grant No.5217002131A).

## Conflict of interest

The authors declare that the research was conducted in the absence of any commercial or financial relationships that could be construed as a potential conflict of interest.

## Publisher’s note

All claims expressed in this article are solely those of the authors and do not necessarily represent those of their affiliated organizations, or those of the publisher, the editors and the reviewers. Any product that may be evaluated in this article, or claim that may be made by its manufacturer, is not guaranteed or endorsed by the publisher.

## Appendix 1: The coding of LREs

A meaning-based and elaborate LRE.

**Table:**

1 Yan:	我感觉他那个, 第三段就完全跟文章的那个要求不一样，我不知道为什么。
	I feel that the third paragraph is completely different from what is required by the prompt, but I do not why I have this feeling.
2 Zhi:	你觉得哪里不一样?
	What do you think is different?
3 Yan:	那个题目里面说的是, 你觉得什么可以鼓励年轻人去做户外运动，但是…
	The prompt says, “what might encourage young people to do outdoor activities,” but…
4 Zhi:	就感觉没怎么说很具体的方法。
	It seems that (the writer) does not mention any specific measures.
5 Yan:	对, 他一直在说年轻人应该怎么做, 但是他没有说什么可以鼓励他们。
	Yes, he is talking about what young people should do rather than what might encourage them.
6 Zhi:	对, 我也觉得。
	Yes, I think so.

A form-based and limited LRE

**Table:**

1 Su:	那你觉得这个 first and foremost 要改吗?
	Do you think “first and foremost” should be replaced?
2 Jin:	啊?
	Huh?
3 Su:	你觉得这三个衔接词都要改吗?
	Do you think all the three transition words should be replaced?
4 Jin:	正在想。
	I’m thinking. (no response afterward)

## References

[ref1] Alshuraidah A. Storch N. (2019). Investigating a collaborative approach to peer feedback. ELT J. 73, 166–174. doi: 10.1093/elt/ccy057

[ref2] Baralt M. Gurzynski-Weiss L. Kim Y. (2016). “Engagement with language: how examining learners’ affective and social engagement explains successful learner-generated attention to form” in Peer interaction and second language learning: Pedagogical potential and research agenda. eds. Sato M. Ballinger S. (Amsterdam: John Benjamins), 209–239.

[ref3] Berggren J. (2015). Learning from giving feedback: a study of secondary-level students. ELT J. 69, 58–70. doi: 10.1093/elt/ccu036

[ref4] Chang C. Y. (2016). Two decades of research in L2 peer review. J. Writing Res. 8, 81–117. doi: 10.1558/wap.32515

[ref5] Chen X. Lee I. (2022). Conflicts in peer interaction of collaborative writing – a case study in an EFL context. J. Second. Lang. Writ. 58:100910. doi: 10.1016/j.jslw.2022.100910

[ref6] Chen W. Yu S. (2019). A longitudinal case study of changes in students’ attitudes, participation, and learning in collaborative writing. System 82, 83–96. doi: 10.1016/j.system.2019.03.005

[ref01] Dao P. (2019). Effects of task goal orientation on learner engagement in task performance. International Review of Applied Linguistics in Language Teaching 59, 315–334. doi: 10.1515/iral-2018-0188

[ref7] Dao P. McDonough K. (2018). Effect of proficiency on Vietnamese EFL learners’ engagement in peer interaction. Int. J. Educ. Res. 88, 60–72. doi: 10.1016/j.ijer.2018.01.008

[ref8] Dao P. Nguyen M. X. N. C. Duong P. Tran-Thanh V. (2021). Learners’ engagement in L2 computer-mediated interaction: chat mode, interlocutor familiarity, and text quality. Mod. Lang. J. 105, 767–791. doi: 10.1111/modl.12737

[ref9] De Guerrero M. C. M. Villamil O. S. (2000). Activating the ZPD: mutual scaffolding in L2 peer revision. Mod. Lang. J. 84, 51–68. doi: 10.1111/0026-7902.00052

[ref10] Diab N. M. (2010). Effects of peer- versus self-editing on students’ revision of language errors in revised drafts. System 38, 85–95. doi: 10.1016/j.system.2009.12.008

[ref11] Donato R. (1988). Beyond group: A psycholinguistic rationale for collective activity in second-language learning. Doctoral dissertation. Newark: University of Delaware.

[ref12] Fan Y. Xu J. (2020). Exploring student engagement with peer feedback on L2 writing. J. Second. Lang. Writ. 50:100775. doi: 10.1016/j.jslw.2020.100775

[ref13] Fredricks J. Blumenfeld P. Paris A. (2004). School engagement: potential of the concept, state of evidence. Rev. Educ. Res. 74, 59–109. doi: 10.3102/00346543074001059

[ref14] Han Y. Hyland F. (2015). Exploring learner engagement with written corrective feedback in a Chinese tertiary EFL classroom. J. Second. Lang. Writ. 30, 31–44. doi: 10.1016/j.jslw.2015.08.002

[ref15] Helme S. Clarke D. (2001). Identifying cognitive engagement in the mathematics classroom. Math. Educ. Res. J. 13, 133–153. doi: 10.1007/BF03217103

[ref16] Hu G. (2005). Using peer review with Chinese ESL student writers. Lang. Teach. Res. 9, 321–342. doi: 10.1191/1362168805lr169oa

[ref17] Hyland K. Hyland F. (2006). Feedback in second language writing: Contexts and issues. New York: Cambridge University Press.

[ref18] Kamimura T. (2006). Effects of peer feedback on EFL student writers at different levels of English proficiency: a Japanese context. TESL Canada J. 23, 12–39. doi: 10.18806/tesl.v23i2.53

[ref02] Kim Y. McDonough K. (2008). The effect of interlocutor proficiency on the collaborative dialogue between Korean as a second language learners. Language Teaching Research 12, 211–234.

[ref19] Lambert C. Philp J. Nakamura S. (2017). Learner-generated content and engagement in second language task performance. Lang. Teach. Res. 21, 665–680. doi: 10.1177/1362168816683559

[ref20] Liu J. Hansen J. (2002). Peer response in second language writing classrooms. Ann Arbor, MI: University of Michigan Press.

[ref21] Lundstrom K. Baker W. (2009). To give is better than to receive: the benefits of peer review to the reviewer’s own writing. J. Second. Lang. Writ. 18, 30–43. doi: 10.1016/j.jslw.2008.06.002

[ref22] Min H. T. (2005). Training students to become successful peer reviewers. System 33, 293–308. doi: 10.1016/j.system.2004.11.003

[ref23] Nakamura S. Phung L. Reinders H. (2021). The effect of learner choice on L2 task engagement. Stud. Second. Lang. Acquis. 43, 428–441. doi: 10.1017/S027226312000042X

[ref24] Nelson G. L. Carson J. G. (1998). ESL students’ perceptions of effectiveness in peer response groups. J. Second. Lang. Writ. 7, 113–131. doi: 10.1016/S1060-3743(98)90010-8

[ref25] Philp J. Duchesne S. (2016). Exploring engagement in tasks in the language classroom. Annu. Rev. Appl. Linguist. 36, 50–72. doi: 10.1017/S0267190515000094

[ref26] Phung L. (2017). Task preference, affective response, and engagement in L2 use in a US university context. Lang. Teach. Res. 21, 751–766. doi: 10.1177/1362168816683561

[ref27] Qiu X. Lo Y. Y. (2017). Content familiarity, task repetition and Chinese EFL learners’ engagement in second language use. Lang. Teach. Res. 21, 681–698. doi: 10.1177/1362168816684368

[ref28] Schunk D. H. Mullen C. A. (2012). “Self-efficacy as an engaged learner” in Handbook of research on student engagement. eds. Christenson S. L. Reschly A. L. Wylie C. (New York, NY: Springer), 219–235.

[ref29] Sinatra G. M. Heddy B. C. Lombardi D. (2015). The challenges of defining and measuring student engagement. Educ. Psychol. 50, 1–13. doi: 10.1080/00461520.2014.1002924

[ref30] Storch N. (2001). An investigation into the nature of pair work in an ESL classroom and its effect on grammatical development. Doctoral dissertation. Melbourne: University of Melbourne.

[ref31] Storch N. (2002). Patterns of interaction in ESL pair work. Lang. Learn. 52, 119–158. doi: 10.1111/1467-9922.00179

[ref32] Storch N. (2008). Metatalk in a pair work activity: level of engagement and implications for language development. Lang. Aware. 17, 95–114. doi: 10.1080/09658410802146644

[ref33] Storch N. Aldosari A. (2013). Pairing learners in pair work activity. Lang. Teach. Res. 17, 31–48. doi: 10.1177/1362168812457530

[ref34] Svalberg A. M. L. (2009). Engagement with language: interrogating a construct. Lang. Aware. 18, 242–258. doi: 10.1080/09658410903197264

[ref35] Swain M. Lapkin S. (1998). Interaction and second language learning: two adolescent French immersion students working together. Mod. Lang. J. 82, 320–337. doi: 10.1111/j.1540-4781.1998.tb01209.x

[ref36] Tan L. L. Wigglesworth G. Storch N. (2010). Pair interactions and mode of communication: comparing face-to-face and computer mediated communication. Australian review of. Appl. Linguis. 33:27. doi: 10.2104/aral1027

[ref37] Tsui A. B. M. Ng M. M. Y. (2000). Do secondary L2 writers benefifit from peer comments? J. Second. Lang. Writ. 9, 147–170. doi: 10.1016/S1060-3743(00)00022-9

[ref38] van den Bos A. H. Tan E. (2019). Effects of anonymity on online peer review in second-language writing. Comput. Educ. 142:103638. doi: 10.1016/j.compedu.2019.103638

[ref39] Villamil O. S. De Guerrero M. C. M. (1996). Peer revision in the L2 classroom: social-cognitive activities, mediating strategies, and aspects of social behavior. J. Second. Lang. Writ. 5, 51–75. doi: 10.1016/S1060-3743(96)90015-6

[ref40] Wang W. (2014). Students’ perceptions of rubric-referenced peer feedback on EFL writing: a longitudinal inquiry. Assess. Writ. 19, 80–96. doi: 10.1016/j.asw.2013.11.008

[ref41] Watanabe Y. (2008). Peer-peer interaction between L2 learners of different proficiency levels: their interactions and reflections. Can. Modern Lang. Rev. 64, 605–635. doi: 10.3138/cmlr.64.4.605

[ref42] Watanabe Y. Swain M. (2007). Effects of proficiency differences and patterns of pair interaction on second language learning: collaborative dialogue between adult ESL learners. Lang. Teach. Res. 11, 121–142. doi: 10.1177/136216880607074599

[ref43] Yu S. Lee I. (2015). Understanding EFL students’ participation in group peer feedback of L2 writing: a case study from an activity theory perspective. Lang. Teach. Res. 19, 572–593. doi: 10.1177/1362168814541714

[ref44] Yu S. Zhang Y. Yao Z. Yuan K. Zhang L. (2019). Understanding student engagement with peer feedback on master’s theses: a Macau study. Assess. Eval. High. Educ. 44, 50–65. doi: 10.1080/02602938.2018.1467879

[ref45] Zaccaron R. Xhafaj D. C. (2020). Knowing me, knowing you: a comparative study on the effects of anonymous and conference peer feedback on the writing of learners of English as an additional language. System 95, 102367–102313. doi: 10.1016/j.system.2020.102367

[ref46] Zhang B. (2021). Engaging in dialogue during collaborative writing: the role of affective, cognitive, and social engagement. Lang. Teach. Res.:136216882110540. doi: 10.1177/13621688211054047

[ref47] Zheng Y. Yu S. (2018). Student engagement with teacher written corrective feedback in EFL writing: a case study of Chinese lower-proficiency students. Assess. Writ. 37, 13–24. doi: 10.1016/j.asw.2018.03.001

[ref48] Zhu W. Mitchell D. (2012). Participation in peer response as activity: an examination of peer response stances from an activity theory perspective. TESOL Q. 46, 362–386. doi: 10.1002/tesq.22

